# Laparoscopic radical nephrectomy versus open radical nephrectomy in T1-T3 renal tumors: An outcome analysis

**DOI:** 10.4103/0970-1591.38602

**Published:** 2008

**Authors:** Arvind P. Ganpule, Rajan Sharma, Manohar Thimmegowda, Muthu Veeramani, Mahesh R. Desai

**Affiliations:** Department of Urology, Muljibhai Patel Urological Hospital, Nadiad - 387 001, Gujarat, India

**Keywords:** Carcinoma, laparoscopic, nephrectomy

## Abstract

**Aims:**

To compare laparoscopic radical nephrectomy (LRN) with open radical nephrectomy (ORN) in T1-T3 renal lesions.

**Materials and Methods:**

The records of 65 patients who underwent LRN between January 2002 and December 2006 were entered prospectively in a database. The patients were compared with 56 patients who had undergone ORN between January 2000 and December 2005. The two groups were comparable in terms of age, body mass index (BMI) and tumor size. LRN was compared with ORN in terms of operative room time, blood loss, *complications*, analgesic requirement, hospital stay and start of oral intake. The oncologic efficacy was evaluated in stages T1 and T2 in terms of cancer-free and overall survival.

**Results:**

The laparoscopy group had a significantly shorter hospital stay (5.72, range 3-23 days vs. 9.18, range 4-23 days, *p* value: < 0.0001), analgesia requirement (175.65, range 50-550 mg vs. 236, range 0-1100 mg of tramadol, *p* value: < 0.03), hemoglobin decline (1.55, range 0.1 to 4.4 mg/dl vs. 2.25, range 0.2 - 7 mg/dL, *p* value: < 0.001) and hematocrit drop (4.83, range 0.3 - 12.9 vs. 7.06 range 2 -18, *p* value: < 0.0001). The majority of specimens showed renal cell carcinoma. In the laparoscopy group, 29 tumors were T1 stage, 18 were T2, while eight were T3. In the open surgery group, 25 tumors were T1, 19 were T2 and 12 were T3. The cancer-free survival rate at 24 months for ORN and LRN in T1 lesions was 91.7% and 93.15% respectively and the patient survival rate was 100% in both groups. The cancer-free survival rate at 24 months for ORN and LRN in T2 lesions was 88.9% and 94.1%, respectively and the patient survival was 100% and 94%, respectively. After LRN, there was one instance of port site metastasis, local recurrence and distant metastasis. All recurrences were distant after ORN.

**Conclusion:**

Laparoscopic radical nephrectomy has advantages in terms of shorter hospitalization and a lower analgesia requirement. It is feasible and produces effective cancer control in T1 lesions, comparable to that of its open counterpart in T2 and selected cases of T3 lesions.

## INTRODUCTION

Clayman and associates first described laparoscopic radical nephrectomy (LRN) at Washington University in June 1990.[1] The aim of laparoscopy has been to duplicate the principles of open radical nephrectomy (ORN) in terms of oncologic efficacy.[2] Recent reports suggest that LRN can be done with comparable oncologic outcomes even in larger tumors (Stage T2) as a surgeon ascends the learning curve.[2,3] We report our experience with LRN and compare the outcome with that of ORN in terms of safety, morbidity and oncologic outcome.

## MATERIALS AND METHODS

### Study population

The records of 65 patients who underwent LRN between January 2002 and December 2006 were compared with those of 56 patients who underwent ORN between January 2000 and December 2005. All patients had an ultrasound scan of the abdomen with a contrast-enhanced CT scan preoperatively. Metastatic workup included a chest radiograph and liver function tests. The patients were staged according to the International TNM Staging system for Renal cell carcinoma[4] and followed up accordingly.[5] Laparoscopic radical nephrectomy was performed by either a transperitoneal or a retroperitoneal approach. The criterion for selecting transperitoneal and reteroperitoneal approach was dependent on surgeon's preference.

### Surgical technique

Transperitoneal LRN was performed using three or four ports. The dissection commenced by incising the white line of Toldt and reflecting the colon. The ureter was identified and lifted off the psoas muscle. The dissection proceeded outside Gerota's fascia toward the lower pole. The lumbar vein and adrenal vein were doubly clipped with Allport™ clips and cut. The renal vein was dissected free. The renal artery was bared and doubly secured with Hem-o-lok™ clips. Adrenalectomy was done in all upper-pole tumors and T2 lesions.[6] The specimen was retrieved through an incision in the right iliac fossa or a Pfannensteil incision.

Retroperitoneal LRN was done by creating a space at the tip of the 12^th^ rib with a combination of blunt and balloon dissection. The working port was placed at a point between the midaxillary line and the anterior axillary line (5 cm above the iliac crest). A 5-mm port was inserted at the junction of the 12^th^ rib and paraspinal muscles (renal angle). The dissection was kept outside Gerota's fascia and the renal vessels were clipped with Hem-o-lok clips.

Open radical nephrectomy was done employing a flank incision. All were done retroperitoneal. We do not perform lymphadenectomy.

### Outcome analysis

The safety and efficacy of LRN was compared with the open technique. The parameters compared were operative room time (ORT), hematocrit drop, analgesia required, complications, hospitalization time and time to start of oral intake. The oncologic efficacy was evaluated by comparing local and distant recurrence, surgical margin status and survival using Kaplan-Meier analysis. The oncologic efficacy was compared separately for Stages T1, T2 and T3 using Student's t-test.

## RESULTS

In the laparoscopy group, 29 tumors were Stage T1, 18 were T2, while eight were T3. In the open surgery group, 25 tumors were Stage T1, 19 were T2 and 12 were T3. The staging was done clinically and confirmed on histopathology. The diagnosis of T3 lesions was based on clinical suspicion, confirmed on pathology. In our series the clinical diagnosis correlated with histopathology in 63% of patients in the laparoscopic group while in the open group it correlated in 42%. The two groups were comparable in terms of age, height, weight, BMI, specimen weight and size. Retrieval bag was used in the last 20 cases.

All four open conversions in the LRN group were in the first 30 cases. Three of them were attributable to difficulty in progression and one to renal vein bleeding. The complications in our series were comparable in both groups. The complications encountered in the LRN group were renal vein bleeding (n = 1), port site infection (n = 3), chest infection (n = 1) and subacute intestinal obstruction (n = 1).

The complications encountered in the ORN group were IVC injury (n = 1), wound infection (n = 1), Pneumothorax (n = 2) and Pleural injury (n = 1).

The laparoscopy group had a significantly shorter hospital stay, a lower analgesia requirement and less hemoglobin and hematocrit drop [Table 1].

**Table 1:** Characteristics of patients undergoing radical nephrectomy and operative data

	Laparoscopy	Open	*P* value
No. of patients	65	56	
Mean age (years)	52.96 (22-80)	52 (2-80)	0.38
BMI	25.10 ± 4.58	24.45 ± 5.67	0.23
M/F	50/15	43/13	
Side (L/R)	32/33	33/23	
Mean ASA score	1.75	2.00	0.49
Mean OR time (min)	179.50 (60-360)	158.6 (60-330)	0.02*
Hemoglobin drop (g/dL)	1.55 (0.1-4.4)	2.25 (0.2-7)	0.001*
Hematocrit drop	4.83 (0.3-12.9)	7.06 (2-18)	<0.0001*
Specimen weight	575.15 (120-1400)	583 (150-2500)	0.45
Size	7.14 (2-17)	8.05 (3-18)	0.07
Mean analgesia (mg of tramadol)	175.65 (50-550)	236 (0-1100)	0.03*
Mean hospital stay (days)	5.72 (3-23)	9.18 (4-23)	<0.0001*
Mean time to start of oral intake (h)	24.7 (20-48)	29.2 18-120)	0.02*

Demographic profile

There was no difference in the outcome of T1 and T2 lesions [Table 2].

**Table 2:** Comparison of results for T1 and T2 tumors (Laparoscopy group)

	T1	T2	*P* value
No. of patients	29	18	
Mean age (years)	51.66 (22-75)	55.22 (30-80)	0.20
BMI	25.13 ± 3.74	23.81 ± 5.07	0.16
M/F	22/7	15/3	
Side (L/R)	9/20	13/5	
Mean ASA	1.79	1.70	0.49
Mean OR time	168.44 (60-300)	187.94 (120-360)	0.13
Hemoglobin drop (g/dL)	1.46 (0.1-2)	1.84 (0.3-4.4)	0.06
Hematocrit drop	4.61 (0.3-7)	5.65 (1-10.9)	0.08
Specimen weight (g)	455.13 (120-910)	748.33 (366-1400)	<0.0001*
Size (cm)	4.79 ± 1.29	10.26 ± 2.5	<0.0001*
Mean analgesia (mg of tramadol)	167.5 (50-550)	164.7 (50-450)	0.47
Mean hospital stay (days)	5.55 (3-23)	5.58 (3-12)	0.48
Mean time to start of oral intake (h)	22.7 ± 2.33	26 ±7.41	0.02*

Comparative analysis

Twenty-three per cent of patients (n = 15) received blood transfusions in the LRN group and 41% (n = 23) of patients in the ORN group required a blood transfusion. Hemoglobin assessment was done at 48 h postoperatively.

**Figure 1:**
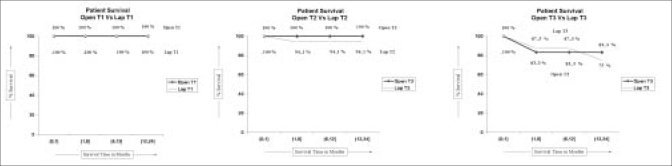
Kaplan Meier analysis of patient survival for open versus laparoscopic approach in T1, T2, T3 tumours

**Figure 2:**
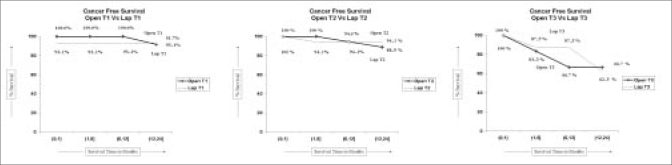
Kaplan Meier analysis of cancer free survival for open versus laparoscopic approach in T1, T2, T3 tumours

The oncologic efficacy was evaluated by comparing local and distant recurrence, surgical margin status and survival using Kaplan-Meier analysis. The cancer-free survival rate at 24 months for ORN and LRN in T1 lesions was 91.7% and 93.15%, respectively and the patient survival rate was 100% in both groups. The cancer-free survival rate at 24 months for ORN and LRN in T2 lesions was 88.9% and 94.1%, respectively. The patient survival rate was 100% and 94%, respectively. The cancer-free survival rate at 24 months for ORN and LRN in T3 lesions was 66.7% and 62.5%, respectively and the patient survival was 83.3% and 75%, respectively [Figures 1, 2].

One of the patients had all three types of recurrences i.e. local, port site and distant metastases, while one patient had local metastases and another had distant metastases [Table 3].

**Table 3:** Local and distant recurrences

**Laparoscopy group**						

Case No.	Size (cm)	Histopathology	Time to recurrence	Type of recurrence		
				
				Local recurrence	Port site metastases	Distant metastases

26	4 × 4	Papillary RCC, capsule infiltrated	Two years	Yes	Yes	Yes
39	9 × 4	Well-differentiated, sarcomatoid changes	Eight months	Yes	No	No
59	10 × 4	Focal sarcomatoid diff; 60% tumor necrotic, grade 3	Two months	No	No	Yes

**Table:** 

**Open group**					

**Case No.**	**Size (cm)**	**Margins**	**Histopathology**	**Time to recurrence**	**Type of recurrence**

2	7 × 6	Negative	Papillary RCC	Three months	Pulmonary metastases
14	6 × 6	Negative	Squamous cell carcinoma	18 months	Pulmonary mets
23	6 × 4	Negative	Squamous cell carcinoma	24 months	Pulmonary mets with nodal mets

The upper size limit for LRN has been considered to be 13 cm.[4] We have operated on a lesion of 15 cm. As our experience increased, the acceptable specimen size went on increasing and simultaneously the conversion rate decreased [Table 4].

**Table 4:** Impact of experience on laparoscopic radical nephrectomy

	1-10	11-20	21-30	31-40	41-50	51-60	61 till date
Number	10	10	10	10	10	10	5
Operating time (min)	175.7	169	143.3	165	184.5	212.5	224
Hb drop (g%)	1.9	1.4	1.8	1.4	1.3	1.7	1.1
PCV drop	6.5	4.7	6.5	4.1	3.7	5.0	2.6
Specimen weight (kg)	511	419	563	632	542.6	709.8	722.2
Tumor size (cm)	6.5	6.05	7.8	7.6	5.9	8.9	7.4
Analgesia (mg)	168.7	109	105	250	210	145	320
Time to oral intake (h)	27	23.1	25.8	25.4	24.6	23.4	23.6
Hospital stay (days)	4.75	7.2	3.8	5.3	4.9	7.3	7.0
Complications							
Minor	1	-	1	-	-	-	-
Major	1	1		1	-	-	-
Conversion	2	1	1	1	-	-	-

## DISCUSSION

Laparoscopic radical nephrectomy is now a widely practiced and accepted treatment modality for T1 lesions.[2] The aim of laparoscopy has been to duplicate the principles of open radical nephrectomy (ORN) in terms of oncologic efficacy.[2]

Initial studies relied on the surgical margin status and the specimen weight to assess oncologic efficacy. These studies suggested that the specimen weight should be equivalent to preoperative size or 20% less if removed by morcellation.[7] In our study, the specimen weight was equivalent to that of ORN. The margins were positive in two cases, one in a patient with T3A disease and the other in a patient with T3B disease. Our experience suggests that renal vein involvement and IVC involvement are technically more challenging and increase the chance of conversion and recurrence. We feel that T3 tumors with only perinephric involvement can be selected for laparoscopic approach.

Local recurrence is defined as evidence of recurrent disease in the renal fossa.[8,9] Portis and associates[8] demonstrated five-year recurrence-free survival and cancer-specific survival rates of 92% and 96%, respectively. Although the follow-up is short our study shows similar results on cancer-specific and patient survival at 24 months [Figures 1, 2]. One of the patients had all three types of recurrences i.e. local, port site and distant metastases, while there was one instance of local metastases and a distant metastases. All recurrences after ORN were distant [Table 3].

In urology, initial reports of port-site recurrence followed laparoscopic lymphadenectomy for carcinoma of the prostate or bladder.[10] The measures suggested to reduce port-site recurrence are use of a bag for intact removal of specimen and appropriate precautions before morcellation such as redraping and irrigating to prevent tumor contamination.[11] Dhobada *et al.*, have reported a port-site recurrence eight months after LRN for a T2N0M0 RCC. The specimen was retrieved by an Endocatch bag.[12] A recent report highlights the role of tumor and host biology in port-site metastasis.[13] In our series, we had one instance of port-site recurrence. The specimen in this case was retrieved by manual extraction. The patient presented with a lump at the site of the 11-mm port.

As surgeons ascend the learning curve, they become comfortable operating on large tumors. The upper size limit for LRN has been considered to be 13 cm.[8,14] We have operated on a lesion of 15 cm. As our experience increased, the acceptable specimen size went on increasing and simultaneously the conversion rate decreased. The stay was longer as the majority of patients were traveling from long distance and the patient had longer stay on request. Moreover as our experience increased we have been operating on larger tumors and hence the longer stay [Table 4].

Although it is not done at our institute, specimen morcellation has been practiced at other centers, the cited advantage being less pain, faster convalescence and shorter incisions. The perceived disadvantages are difficulties in histopathologic analysis and the risk of tract seeding.[15] In our series, we retrieved the specimen intact and since we had the instance of port-site metastasis, we removed the specimen with a homemade bag.

Whether one uses a transperitoneal or a retroperitoneal approach generally depends on surgeon preference and comfort level. When performed in accordance with oncologic principles such as early control of the renal hilum and en bloc dissection of the kidney outside Gerota's fascia, the outcomes of both should be comparable. The advantages of the transperitoneal approach include familiarity of the anatomy and a good working space. The disadvantages include the need to reflect the abdominal structures. The retroperitoneal approach offers rapid access to the hilum and avoidance of the peritoneal cavity. In theory, this should reduce the incidence of postoperative ileus and injury to intraperitoneal contents.[16] The majority of our LRNs have been done with the transperitoneal approach, as we are comfortable with it.

## CONCLUSION

Laparoscopic radical nephrectomy has advantages in terms of shorter hospitalization and a lower analgesia requirement. It is feasible and produces effective cancer control in T1 lesions, comparable to that of its open counterpart in T2 and selected cases of T3 lesions. Reports of port-site metastasis mandate a multicenter analysis.
